# FEM Analysis of Sezawa Mode SAW Sensor for VOC Based on CMOS Compatible AlN/SiO_2_/Si Multilayer Structure

**DOI:** 10.3390/s18061687

**Published:** 2018-05-24

**Authors:** Muhammad Zubair Aslam, Varun Jeoti, Saravanan Karuppanan, Aamir Farooq Malik, Asif Iqbal

**Affiliations:** 1 Department of Electrical and Electronics Engineering, Universiti Teknologi PETRONAS, 32610 Seri Iskandar, Perak, Malaysia; varun_jeoti@utp.edu.my (V.J.); aamir.malik@utp.edu.my (A.F.M.); asif.iqbal@utp.edu.my (A.I.); 2 Department of Mechanical Engineering, Universiti Teknologi PETRONAS, 32610 Seri Iskandar, Perak, Malaysia; saravanan_karuppanan@utp.edu.my

**Keywords:** AlN, FEM simulation, Sezawa mode, VOC

## Abstract

A Finite Element Method (FEM) simulation study is conducted, aiming to scrutinize the sensitivity of Sezawa wave mode in a multilayer AlN/SiO_2_/Si Surface Acoustic Wave (SAW) sensor to low concentrations of Volatile Organic Compounds (VOCs), that is, trichloromethane, trichloroethylene, carbon tetrachloride and tetrachloroethene. A Complimentary Metal-Oxide Semiconductor (CMOS) compatible AlN/SiO_2_/Si based multilayer SAW resonator structure is taken into account for this purpose. In this study, first, the influence of AlN and SiO_2_ layers’ thicknesses over phase velocities and electromechanical coupling coefficients (*k*^2^) of two SAW modes (i.e., Rayleigh and Sezawa) is analyzed and the optimal thicknesses of AlN and SiO_2_ layers are opted for best propagation characteristics. Next, the study is further extended to analyze the mass loading effect on resonance frequencies of SAW modes by coating a thin Polyisobutylene (PIB) polymer film over the AlN surface. Finally, the sensitivity of the two SAW modes is examined for VOCs. This study concluded that the sensitivity of Sezawa wave mode for 1 ppm of selected volatile organic gases is twice that of the Rayleigh wave mode.

## 1. Introduction

The monitoring of volatile organic compounds (VOCs) is very crucial in various application fields like in industrial safety, fire detection, indoor air quality and health monitoring [1,2]. VOCs are hazardous air pollutants because of their toxic characteristics [3]. Being exposed to VOCs for a short period of time can cause the human nervous system to be badly affected with symptoms like nausea, headaches, visual illnesses, allergies and respiratory tract itching. It can cause permanent damage if one remains unmasked for a considerably long time. Thus, the monitoring and detection of VOCs at an early stage is essential. VOCs evaporate even at room temperature [3]. The source of emittance of VOCs can be fuel, vehicle smoke, industrial sources and paints/cleaning reagents.

The existing monitoring systems for VOCs are usually based on infrared spectroscopy, gas chromatography and mass spectrometry. Although these approaches provide good sensitivity and selectivity, they are not very suitable for real time monitoring [3], as these technologies are comprised of sensitive electronic apparatuses and need carrier gas flow throughout the operation. Conversely, Surface Acoustic Wave (SAW) sensors, which have received great attention of late, are quite suitable for real time monitoring. SAW sensors are popular because of their real time detection capability, simple fabrication procedure, small size, excellent repeatability, high sensitivity and low cost [4]. Other than gas sensing applications, the SAW devices also serve a wide range of other applications, such as temperature sensor [5], pressure sensor [6] and mass detector [7] and so forth. Moreover, currently, SAW bio-sensors and microfluidics [8] are also gaining attention for point-of-care health monitoring. For example, in health monitoring, SAW biosensors are being used for the identification of the hepatitis B antibody as SAW sensors are more sensitive and they have a quick response time [9].

In SAW gas sensors, a thin film sensing layer coating is required over the acoustic path. This thin film acts as a mass loading on the SAW device, which causes a change in acoustic velocity and thus a change in the resonant frequency of the device. When a sensing layer coated SAW gas sensor is exposed to different gas concentrations, gas molecules are physically adsorbed at the sensing layer. The amount of change in the center frequency to gas concentration reflects the sensitivity of the sensor. The SAW based gas sensors have been reported in recent literature. A lithium niobate (LiNbO_3_) based SAW resonator as a gas sensor is reported in Reference [10]. A thin PIB layer is coated over LiNbO_3_ to adsorb gas. Moreover, the effect of Interdigital Transducer (IDT) configurations (unidirectional and bidirectional) on a SAW device is also presented. The sensitivity of a SAW resonator is gauged by exposing the sensor to various concentrations of dichloromethane. A room temperature cryptophane-A coated ST-X quartz based SAW methane gas sensor is reported in Reference [11]. The experimental results reveal a fast response with excellent repeatability. Separately, a design of SAW gas sensor with PIB layer over LiNbO_3_ for the detection of VOCs is reported in Reference [12]. The shift in the resonance frequency of the sensor is analyzed when the sensor is exposed to 100 ppm of organic gases. A layered SAW hydrogen gas sensor is developed in Reference [13] by depositing phthalocyanine (Pc) and then palladium (Pd) on the LiNbO_3_ substrate. The designed sensor is able to detect a hydrogen concentration range from 0.5 to 4% in air at about 25 °C. 

According to the existing literature, the majority of SAW gas sensors use a sensing layer that is deposited on single piezoelectric material substrates. The SAW devices have been largely produced with well-known single material substrates, such as lithium niobate (LiNbO_3_), lithium tantalate (LiTaO_3_) and Quartz but single material substrate based SAW devices cannot be grown by CMOS technology. Consequently, the integration of single material SAW devices to silicon integrated circuit processes is incompatible [14]. 

The advancement in the space of SAW fabrication technology has opened the doors for thin film multilayer SAW devices. The thin films of a piezoelectric material deposited on a regular substrate with a high acoustic velocity enable high frequency SAW devices. In last few years, the AlN based multilayer SAW devices have gained the attention of researchers because of the advantage of high acoustic velocity of 5760 m/s as compared to Zinc oxide (ZnO), LiNbO_3_, LiTaO_3_ and quartz. Moreover, AlN has good compatibility to CMOS technologies as it can easily be deposited by physical vapour deposition at low temperatures [15,16].

Literature shows the evidence of many Rayleigh based high frequency devices on AlN/SiO_2_/Si [17,18]. However, there is no evidence of any Sezawa wave mode based sensor in the literature. In [19], a device developed on AlN/diamond is reported with Sezawa mode but that is not CMOS compatible. Similarly, there have been many papers with ZnO/SiO_2_/Si and ZnO/Si with Sezawa mode devices [20,21,22] but none on AlN/SiO_2_/Si. The Sezawa waves exist in layered SAW devices, when a substrate’s SAW velocity is greater than the overlaid piezoelectric film and exhibits higher velocity than Rayleigh mode for same IDTs configurations [23]. As a result, higher resonance frequency is attainable which makes Sezawa mode a potential candidate for future development of high frequency filters, oscillators and sensors. For example, sensors at 2.4 GHz to suit the radio standards for sensor network will be much easier to fabricate using Sezawa mode than Rayleigh Mode. Generally, Sezawa mode exists in so called “slow on fast” structures, which means Sezawa wave can exist in two layers SAW structure, when thin piezoelectric film of lower velocity is deposited on regular bulk substrate (e.g., Diamond as Substrate) of higher velocity. 

This work intends to analyze the Sezawa wave mode, resulting from SiO_2_/Si structure (slow on fast) in AlN/SiO_2_/Si multilayer SAW for gas sensing application. Towards that, we first study the effect of normalized AlN and SiO_2_ thin film thicknesses on proposed SAW device propagation properties (i.e., SAW velocity and electromechanical coupling coefficient) without sensing layer and validate our results with published results. In the second part, we present the mass loading effect of polymer film on an AlN/SiO_2_/Si multilayer SAW device and present the comparative study of both surface modes (Rayleigh and Sezawa) in terms of gas sensitivity. 

## 2. Problem Formulation

*A. Geometry of the Problem*

**Figure 1:**
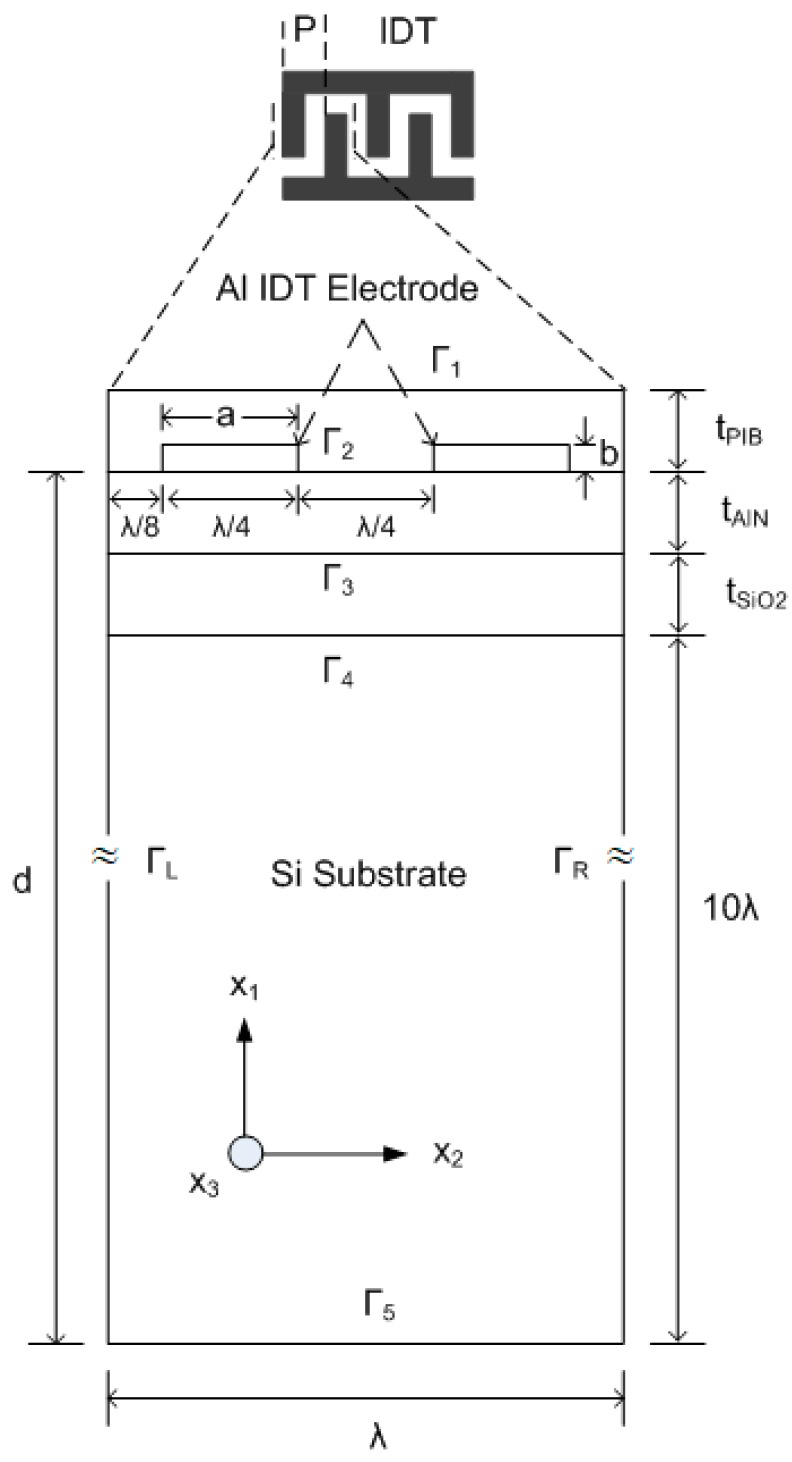
A 2D unit cell geometry used in Finite Element Method (FEM) simulation.

In this work, FEM simulation of a layered SAW device is performed by using COMSOL Multiphysics. The FEM study for different piezoelectric materials has been used, validated and reported by researchers [7,24,25]. In first phase of our study, a 2D FEM model of AlN/SiO_2_/Si SAW resonator structure without a sensing layer is investigated. The purpose of using 2D modelling is to reduce computational complexity. Moreover, a plane strain supposition is used for the solid mechanics. Consequently, the out of plane strain component is zero. The change in the out of plane path can be considered least if the acoustic waves are produced in the plane of the model. The dimensions of the SAW device used in the simulation are summarized in Table 1 and the geometry of the layered SAW resonator is shown in Figure 1.

The IDTs are periodic in nature, therefore to model a SAW resonator, one period of the electrode is enough to model whole resonator. For this purpose, a unit cell of 1λ is considered for the SAW resonator as shown in Figure 1. The depth of the unit cell is chosen as 10λ, as the mechanical displacement depth of the Rayleigh and Sezawa wave modes are most confined at the surface and nearly die out at lower boundary. The metallization ratio is selected as 50% (2a/*λ*) and the relative thickness of Aluminum (Al) electrode (b/*λ*) is chosen as 2.5%, where a and b are the electrodes’ width and height, respectively. The material properties of Al are used from the built-in COMSOL library, that is, a density of 2700 kg/m^3^, Young’s modulus of 70 GPa and Poisson’s ratio of 0.33. The mechanical boundaries of the top and sides of both electrodes are free. The left electrode’s boundaries are set to electrically ground and the right electrode’s boundaries are set to a floating potential with zero surface charge accumulation. The edge effects of the electrode can be ignored as the length of the electrode is much higher than its width. After performing a mesh convergence study, an extremely fine mesh (i.e., maximum automatically generated physics-defined triangular elements) was chosen for all the FEM simulations to get more accurate results. The boundary conditions used in the simulation are summarized in Table 2 and the material constants used in the simulation are summarized in Table 3.

Primarily, the simulations for the Eigen frequency analysis of the layered SAW resonator are performed to analyze the AlN/SiO_2_/Si acoustic velocity and electrochemical coupling coefficient (*k*^2^) for Rayleigh and Sezawa mode as a function of normalized AlN and SiO_2_ thicknesses. The SiO_2_ layer provides electrical isolation between AlN and Si, CMOS compatibility and fast-slow-fast structure. By varying the AlN and SiO_2_ film thicknesses, their effect on the resonance frequency of the SAW resonator is assessed. In general, the acoustic wave velocity is calculated by

(1) $$v=f_{o}\lambda$$

where $f_{o}$ is center frequency and λ is the wavelength of the acoustic wave. In Eigen mode, the value of v is calculated by

(2) $$v=\left(f_{res.}+f_{ant.}\right)p$$

where $f_{res.}$ and $f_{ant.}$ are resonant and anti-resonant frequencies respectively, which are obtained from the Eigen frequency simulation analysis and p is the pitch (electrode width + spacing between electrodes). In our simulation, the value of p = 2 µm (λ/2) throughout all simulations. The value of *k*^2^ for is calculated by [17]

(3) $$k^{2}=2\;\left(\frac{v_{o}-v_{m}}{v_{o}}\right)$$

where $v_{o}$ is free surface velocity with electrically free boundary condition and $v_{m}$ is phase velocity with an electrically shorted boundary condition. The calculated acoustic wave velocity and the electromechanical coupling coefficient as a function of the normalized thickness is presented in next section.

*B. Gas Sensing Phenomena and Equation*

The gas sensing phenomena is simple, when a PIB coated SAW gas sensor is exposed to different gas concentrations; gas molecules are physically adsorbed at the PIB layer. There are two types of adsorption, that is, chemical adsorption and physical adsorption. The chemical adsorption is irreversible whereas physical adsorption makes the process reversible and desorption occurs when the gas leaves. Thus, physical adsorption based sensors exhibit good repeatability [26]. The rate of diffusion of adsorbed gas in the sensing layer defines the response and recovery time. Generally, the thin sensing layer takes less recovery time as the diffused gas molecules rapidly leave the surface upon removing the gas, thus the sensor quickly reaches its equilibrium state [27]. The sensing layer with adsorbed molecules results in an increase of net mass loading over sensor. The partial density of adsorbed gas in PIB thin layer can be calculated as [10].

(4) $$\rho_{gas,PIB}=KMc$$

where the molar mass of the gas is M, K is the PIB/air partition coefficient of the gas and c is the concentration of gas in air. Gas concentration in air can be calculated as [10]

(5) $$c=c_{o}\left(\frac{P}{RT}\right)$$

where $c_{o}$ is the concentration of gas in parts per million (ppm), air pressure is denoted by P, gas constant by R and air temperature by T. Other than density, all effects are neglected. The adsorption of gas is described as a minor rise in the overall density of the PIB film, which can be expressed as

(6) $$\rho =\rho_{PIB}+\rho_{gas,PIB}$$

The above equation gives the total density of PIB after gas adsorption. The resonance frequencies of both surface modes with different heights of PIB film are recorded.

## 3. Results and Discussion

### 3.1. Saw Propagation Analysis

**Figure 2:**
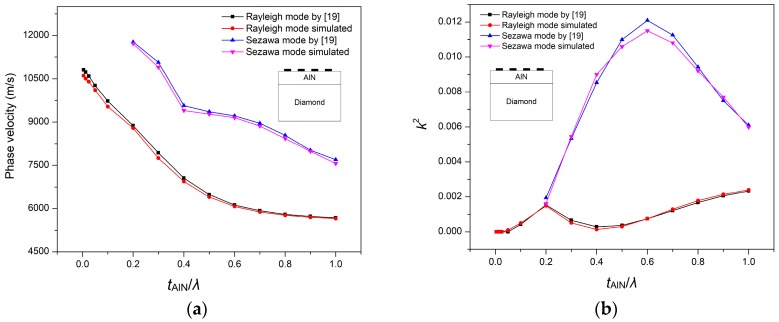
Simulation results (**a**) Phase velocity of Rayleigh causes mass loading effect which is sensing phenomena of and Sezawa mode for AlN/Diamond structure; (**b**) Electromechanical coupling coefficient for Rayleigh and Sezawa wave mode.

**Figure 3:**
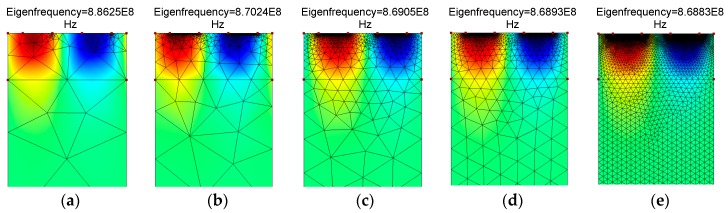
The mesh density profile. (**a**) Triangular elements = 228; (**b**) Triangular elements = 35,485; (**c**) Triangular elements = 49,067; (**d**) Triangular elements = 55,748; (**e**) Triangular elements = 120,503.

**Figure 4:**
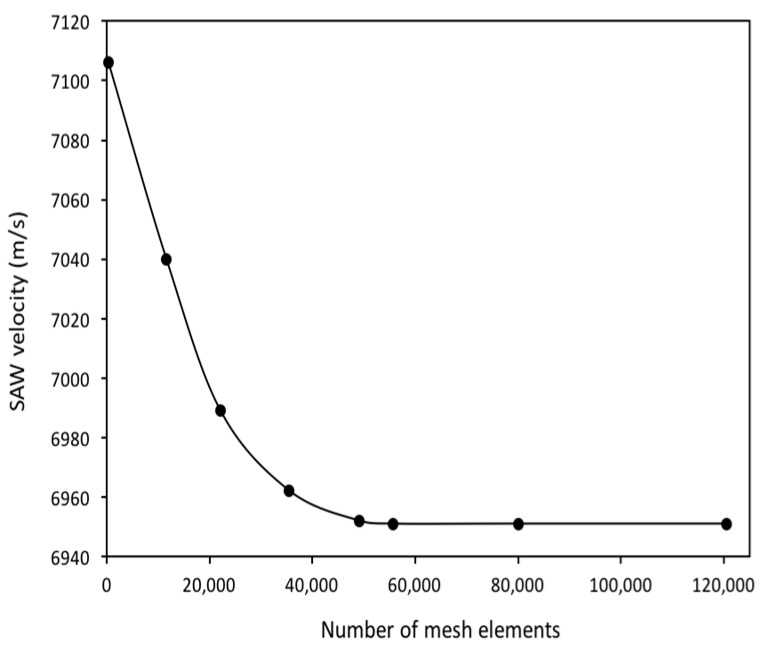
The effect of mesh density on Surface Acoustic Wave (SAW) velocity.

Initially, in order to validate our simulation method for the AlN/SiO_2_/Si (fast-slow-fast) structure, we first used the AlN/Diamond (slow-fast) structure, which was presented in Reference [19]. In that work, the SAW propagation characteristics were theoretically calculated by PC acoustic wave software from McGill University. This software is based on a transfer matrix method for calculating SAW propagation in multilayer structures. We extracted their numerical data by using a semi-automated open source plot digitizer tool, that is, WebPlotDigitizer v. 3.12, which has been used in many published works. In our study, we performed a FEM simulation analysis of the AlN/Diamond structure by using the unit cell as in Figure 1 (but with two layers, i.e., AlN/Diamond) and the boundary conditions as in Table 2. Same material constants were used as described in Reference [19]. The SAW propagation properties are calculated by (1), (2) and (3). The simulated results are shown in Figure 2, which are quite close to that of Reference [19]. The minor error can be attributed to the difference in the methods used. The method used in Reference [19] relied on a transfer matrix method that makes assumptions of several thin layers in approximating the behavior of interfacial layers, while this paper uses an FEM method. In order to justify the sufficiency of mesh density, a mesh convergence study was also conducted until the values of the velocity of SAW became constant. The mesh profile for a specific thickness of the AlN layer (i.e., t_AlN_/λ = 0.4) is summarized in Figure 3. As shown in Figure 4, the SAW velocity becomes constant at 6951 m/s, the value that has been used in this work. It can be mentioned that the velocity, 7056.5 m/s, in Reference [19] is obtained at a much coarser mesh. In order to get accurate results for our study, the maximum number of mesh elements are chosen (i.e., 120,503).

**Figure 5:**
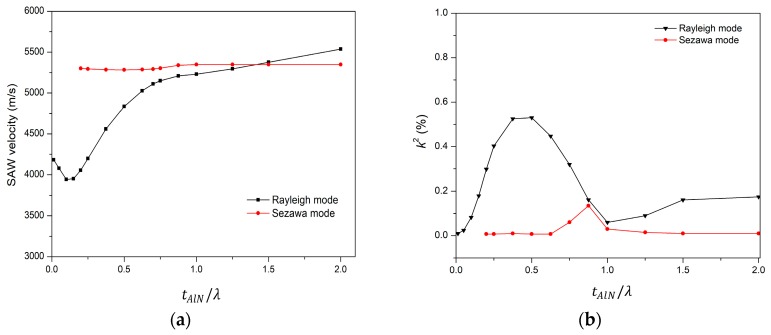

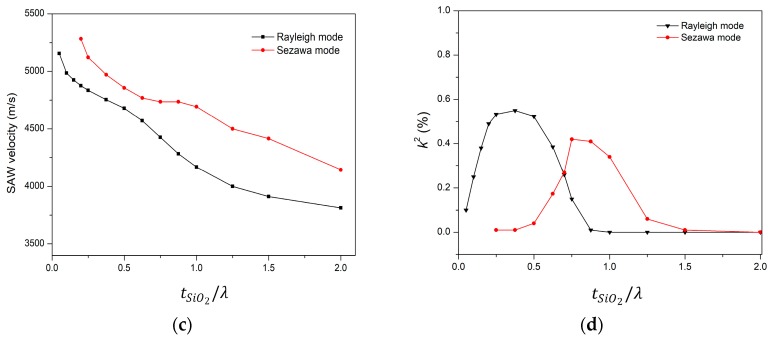
(**a**) Acoustic wave velocity versus normalized thickness of AlN; (**b**) Electromechanical coupling coefficient (*k*^2^) versus normalized AlN thickness; (**c**) Acoustic wave velocity versus normalized thickness of SiO_2_; (**d**) Electromecanical coupling coefficient versus normalized SiO_2_.

**Figure 6:**
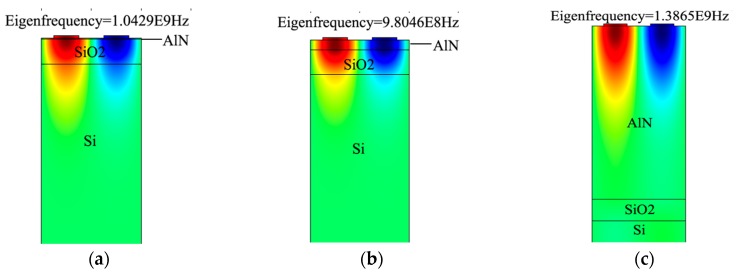
The displacement profile and wave confinement in different layer (Zoom-in view). (**a**) When $t_{AlN}/\lambda \;$ = 0.01. (**b**) When $t_{AlN}/\lambda$ = 0.1 and (**c**) when $t_{AlN}/\lambda$ = 2.

The next simulation is performed to determine the velocity and electromechanical coupling coefficient (*k*^2^) of AlN/SiO_2_/Si structure for the modes polarized in the sagittal plane (Rayleigh type). The typical Sezawa wave mode is generated in a slow-fast structure. On the same principle, in the AlN/SiO_2_/Si (fast-slow-fast) structure, the Sezawa mode is generated by SiO_2_/Si (slow on fast) layers while the piezoelectric AlN layer generates the acoustic waves. The simulation results for the propagation characteristics in the AlN/SiO_2_/Si structure are presented in Figure 5. The phase velocity of Rayleigh mode increases with increasing normalized AlN film thickness ($t_{AlN}/\lambda$), while that of Sezawa mode remains nearly constant. In this simulation, the SiO_2_ thickness is kept constant. In Figure 5a, the value of the phase velocity changes from 4181 m/s to 5537 m/s of Rayleigh mode when 0.01 < $t_{AlN}/\lambda$ < 2 and $t_{SiO2}/\lambda$ is 0.25. For 0.01 < $t_{AlN}/\lambda$ < 0.1, the acoustic velocity in Rayleigh mode decreases from 4181 m/s to 3941 m/s, which suggests that initially the acoustic wave is confined largely in SiO_2_ and also to some degree in Si, as shown in Figure 6a. Therefore, net velocity is the intermediate of SiO_2_ velocities (3750 m/s [28]) and Si (5000 m/s [28]). Similarly, with a further increase in $t_{AlN}/\lambda$, acoustic velocity gradually increases as the acoustic wave starts confining more in the AlN layer and in SiO_2_ and no longer in Si, as shown in Figure 6b. It keeps doing so until the whole acoustic wave is confined only in the AlN substrate (as in Figure 6c), reaching a velocity of 5539 m/s at $t_{AlN}/\lambda$ = 2, close to the theoretical acoustic velocity of AlN (5600 m/s [28]). 

The acoustic velocity in Sezawa mode remains unchanged for 0.01 <$\;\frac{t_{AlN}}{\lambda }$< 2. This is because of the fact that the Sezawa mode depends on the slow-on-fast structure, that is, SiO_2_/Si, which is constant in this case while varying in AlN thickness. In Figure 5b, for the Rayleigh wave, the *k*^2^ firstly increases with an increase in $t_{AlN}/\lambda$ and reaches its maximum value of 0.55% when $t_{AlN}/\lambda$ is 0.5 and then it starts reducing with a further increase of $t_{AlN}/\lambda$. In Sezawa wave mode, the *k*^2^ remains unchanged except for a small peak at $t_{AlN}/\lambda \;$= 0.875, as $t_{SiO2}/\lambda$ = 0.25 is not sufficient to generate Sezawa mode. Next, the AlN layer thickness is kept constant at $t_{AlN}/\lambda$ = 0.5 and the effect of varying $t_{SiO2}/\lambda$ on SAW velocity is analyzed, as shown in Figure 5c.

It is clear that Sezawa mode exhibits a higher acoustic velocity than the Rayleigh mode. For both wave modes, the acoustic wave velocity reduces with increasing SiO_2_ layer thickness, as the acoustic energy starts confining more in the SiO_2_ layer, which has the lowest SAW velocity in the proposed structure. In Figure 5d, for the Rayleigh wave, the *k*^2^ firstly increases with an increase of $t_{SiO2}/\lambda$ and reaches its maximum value of 0.55% when $t_{SiO2}/\lambda$ is 0.375 and then it starts reducing with a further increase of $t_{SiO2}/\lambda$. In Sezawa wave mode, the *k*^2^ initially increases with an increase of $t_{SiO2}/\lambda$ and reaches its maximum value of 0.44% when $t_{SiO2}/\lambda$ = 0.75 and reduces upon a further increase of $t_{SiO2}/\lambda$. In our later study of mass loading sensitivity, we used peak values of *k*^2^ for its relevant mode, which are the optimal points for both modes.

**Figure 7:**
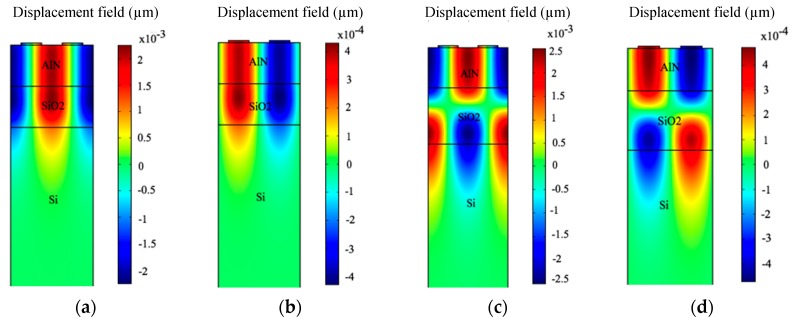
The acoustic wave modes shapes and their y-component of displacement. (**a**) Resonance of Rayleigh mode at 1.167 GHz; (**b**) Anti-resonance of Rayleigh mode at 1.172 GHz; (**c**) Resonance of Sezawa mode at 1.2 GHz. (**d**) Anti-resonance of Sezawa mode at 1.214 GHz.

The displacement profiles of the device are summarized in Figure 7. The mode shapes of the displacement profile are helpful in recognizing the Rayleigh and Sezawa wave modes. The results in Figure 7 are recorded at resonance and anti-resonance modes of Eigen frequency analysis for $t_{AlN}$ = 2 µm and $t_{SiO2}$ = 2.8 µm. The resonance (Figure 7a) and anti-resonance (Figure 7b) modes in the Eigen frequency analysis of Rayleigh mode were observed as 1.167 GHz and 1.172 GHz respectively. Similarly, for Sezawa wave mode, the Eigen frequency for resonance (Figure 7c) and anti-resonance mode (Figure 7d) is recorded at 1.2 GHz and 1.214 GHz respectively.

### 3.2. Analysis of Mass Loading Effect and Gas Sensitivity

#### 3.2.1. Mass Loading Analysis

In the next stage of the study, a thin film of PIB is coated over the surface of the AlN film and the resonance frequency of both modes with different thicknesses of the PIB film is recorded. The PIB is preferred as a sensing film due to its low crystalline property, high permeability, low density and good adhesion properties [3,29]. Moreover, in gas sensing applications, PIB has proven to be more sensitive than other polymers [4]. 

**Figure 8:**
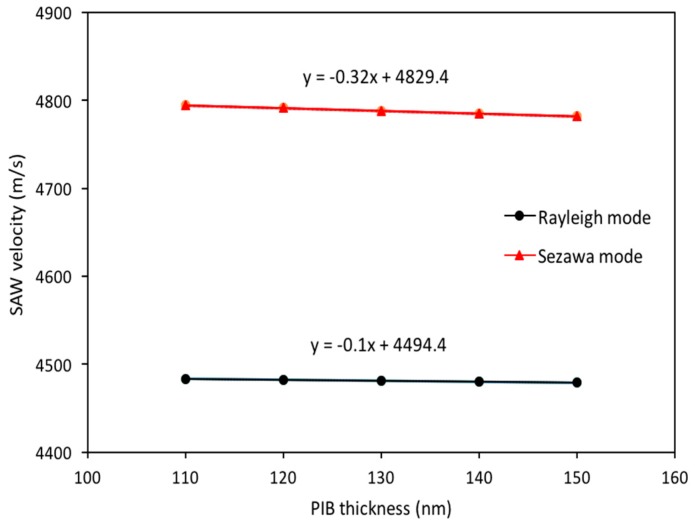
Mass Loading effect on SAW Propagation Velocity.

First, the mass loading sensitivity analysis of the SAW device is performed without exposing PIB to any gas. For this purpose, a thin PIB film is placed over the entire surface of the SAW resonator (as shown in Figure 1). The PIB parameters used in simulation are same as in Reference [12], that is, density as 918 kg/m^3^, Poison’s ratio is 0.48, Young’s modulus is 10 GPa and relative permittivity is 2.2. The thickness of the PIB film ($t_{PIB}$) over the SAW resonator’s surface is varied from 110 nm to 150 nm in steps of 10 nm. The lower limit of $t_{PIB}$ is chosen as 110 nm which is in accordance with the electrode height (100 nm in this case) and the upper limit of $t_{PIB}$ is chosen 150 nm; as attenuation occurs with a further increase in $t_{PIB}$. To study the implications of $t_{PIB}$ on SAW velocity, different thicknesses of PIB are considered and results are shown in Figure 8. It is clear from Figure 8 that the mass loading on the surface of AlN/SiO_2_/Si decreases the acoustic velocity in both SAW modes and the Sezawa mode is more sensitive to surface mass loading as compared to Rayleigh mode. The effect of $t_{SiO2}/\lambda$ and $t_{AlN}/\lambda$ variation on resonant frequencies of Rayleigh ($\Delta f_{R}$) and Sezawa mode ($\Delta f_{s}$) are summarized in Table 4. In this analysis, it is clear that at $t_{SiO2}/\lambda$ = 0.5, Rayleigh mode is almost 2 times more sensitive than Sezawa mode but at $t_{SiO2}/\lambda$ = 0.75, Sezawa mode is more than 3 times more sensitive than Rayleigh mode. It is concluded that an optimal value of sensitivity can be obtained by carefully selecting of $t_{SiO2}/\lambda$ and $t_{AlN}/\lambda$ values.

#### 3.2.2. *Gas* Sensitivity Analysis

The study is extended to analyze the sensitivity of the both surface modes to organic gases. For this purpose, we selected $t_{AlN}/\lambda$ = 0.5, $t_{SiO2}/\lambda \;$= 0.5 for Rayleigh mode and $t_{AlN}/\lambda$ = 0.5, $t_{SiO2}/\lambda$ = 0.75 for Sezawa mode as these appear as the best choices for optimum sensitivity for Rayleigh mode and Sezawa mode independently. The PIB thickness is chosen as 110 nm, to utilize minimum mass loading and fast equilibrium of the sensor. The simulation is performed for selective organic gases that is, trichloromethane, trichloroethylene, carbon tetrachloride and tetrachloroethene. The PIB/air partition coefficient (K), molar mass of gas (M) and partial densities ($\rho_{gas,PIB}$) of adsorbed gases are shown in Table 5. 

**Figure 9:**
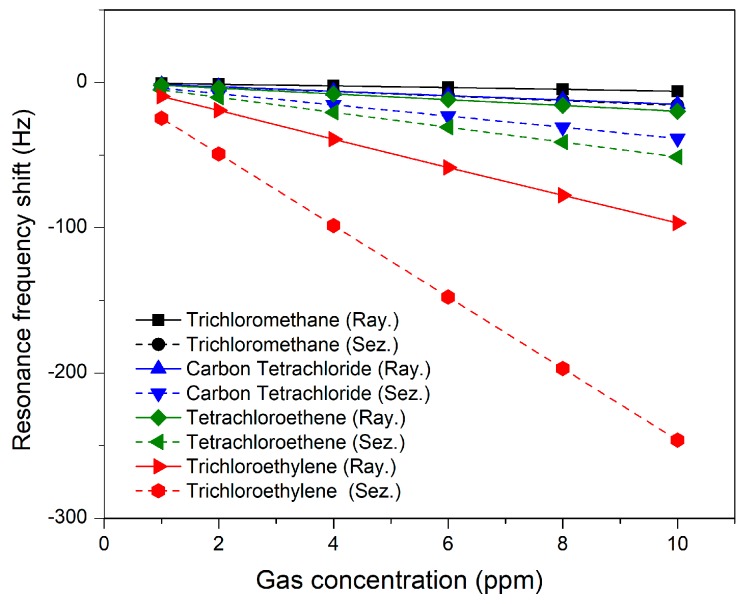
Plot of resonance frequency shift versus gas concentration in ppm.

The measurement is performed for the gas concentrations in the range of 1 ppm to 10 ppm. The selection of the ppm range is made on the basis of the fact that most of the organic gases become hazardous in the range of a few ppm [30]. The results of gas sensing are summarized in Figure 9. The Rayleigh mode $\Delta f_{R}$/ppm and Sezawa mode $\Delta f_{s}$/ppm for trichloromethane, carbon tetrachloride, trichloroethylene and tetrachloroethene are summarized in Table 6. It can be observed that the sensitivity of Sezawa mode for 1 ppm of volatile organic gases is 2 times greater than that of Rayleigh wave mode, so the Sezawa mode SAW sensor proves more sensitive than the Rayleigh mode SAW sensor. The shifts in resonant frequencies are easily detectable with existing circuit topologies. For example, the authors of Reference [31] presented a circuit topology to detect a frequency shift with a resolution of 6.2 mHz. 

The achieved sensitivity of Rayleigh mode in CMOS compatible AlN/SiO_2_/Si based SAW sensor for VOCs is in good agreement with the sensitivity of the Rayleigh mode ZnO/SiO_2_/Si based SAW sensor as in [32]. A ZnO/SiO_2_/Si based SAW sensor is designed to detect VOCs. The polyepichlorohydrine (PECH) is used as a sensing film. The toluene VOC is targeted for analysis. The achieved sensitivity of the sensor is ~2 Hz/ppm with the ZnO film prepared with a 10% O_2_ concentration. However, there is no evidence of the Sezawa mode ZnO/SiO_2_/Si based SAW gas sensor in literature. The results illustrate that the Sezawa mode in AlN/SiO_2_/Si multilayer SAW structure is a good candidate for high sensitive SAW gas sensor applications.

In our targeted application scenario of indoor air quality measurement, we did not require selectivity to a specific gas. In the case of such scenarios where the selectivity of gas is required with a background of interfering gases, a number of methods are possible which are summarized in Reference [1] and are as follows: (i) using a multi-sensor array and pattern recognition; (ii) using an analytical tool like a Gas Chromatography (GC) tube to separate various gases; and (iii) by using a dynamic operation such as temperature cycling. Our proposed methodology will be beneficial in attaining a healthier and safer environment.

## 4. Conclusions

This study is performed to analyze the Sezawa wave mode propagation characteristics in AlN/SiO_2_/Si structure and its potential to be used in gas sensing applications. It is observed that not only does Sezawa mode exist in an AlN/SiO_2_/Si structure but it also exhibits high SAW velocity with a moderate electromechanical coupling coefficient as compared to Rayleigh mode. The selection of AlN and SiO_2_ layer thicknesses is very important for obtaining optimum sensor performance. Moreover, the analysis of PIB mass loading and its sensitivity towards VOCs are analyzed and it is found that Sezawa mode is more sensitive to VOCs than SAW mode. The sensitivity of the Sezawa wave mode to VOCs is shown to be twice that of the Rayleigh wave mode.

## Figures and Tables

**Table 1:** The Summary of dimensions of the device.

Structure Dimensions	Value/µm
Wave length	4 (λ)
Pitch of electrode	2 (λ/2)
Interdigital Transducer (IDT) width	1 (λ/4)
Si substrate thickness	40 (10λ)

**Table 2:** The summary of boundary conditions used in simulation.

Mechanical Boundary Condition		Electrical Boundary Condition
Γ1	Free	Zero Charge/Symmetry
Γ2	Free	Continuity
Γ3	Free	Continuity
Γ4	Free	Continuity
Γ5	Fixed Constraint	Ground
ΓL, ΓR	Periodic Boundary condition	

**Table 3:** The material constants used in simulation.

	Symbol	AlN [24]	SiO_2_ [25]	Si [25]
Density (kg/m^3^)	ρ	3260	2200	2330
Elastic Constants (GPa)	C_11_	345	78.5	166
	C_12_	125	16.1	64
	C_13_	120	16.1	64
	C_33_	395	78.5	166
	C_44_	118	31.2	80
	C_66_	110	31.2	80
Piezoelectric Constants (C/m^2^)	e_15_	−0.48	-	-
	e_31_	−0.45	-	-
	e_33_	1.55	-	-
Dielectric Constant (10^−11^ F/m)	ε_11_	9	3.32	10.62
	ε_33_	11	3.32	10.62

**Table 4:** Summary of mass loading sensitivity at $t_{AlN}/\lambda$ = 0.5.

$t_{SiO2}/\lambda$	Rayleigh Sensitivity (KHz/nm)	Sezawa Sensitivity (KHz/nm)
0.5	40.5	23
0.75	26.42	83.6

**Table 5:** Volatile organic compounds (VOCs) parameters used in simulation and partial densities for 1 ppm.

Gas	K [12]	M (g/mol) [12]	$\rho_{gas,PIB}\;\left(kg/m^{3}\right)$
Trichloromethane	1.927	119.5	0.00041
Carbon Tetrachloride	2.206	153.8	0.00101
Trichloroethylene	2.399	131.4	0.00134
Tetrachloroethene	2.979	165.8	0.00647

**Table 6:** Summary of gas sensitivity analysis.

Analyte	Sensitivity in Rayleigh Mode (Hz/ppm)	Sensitivity in Sezawa Mode (Hz/ppm)
Trichloromethane	0.75	1.57
Carbon Tetrachloride	1.85	3.85
Trichloroethylene	2.53	5.13
Tetrachloroethene	12.1	24.61
